# Writing About Past Failures Attenuates Cortisol Responses and Sustained Attention Deficits Following Psychosocial Stress

**DOI:** 10.3389/fnbeh.2018.00045

**Published:** 2018-03-23

**Authors:** Brynne C. DiMenichi, Karolina M. Lempert, Christina Bejjani, Elizabeth Tricomi

**Affiliations:** ^1^Department of Psychology, Rutgers University, Newark, Newark, NJ, United States; ^2^Department of Psychology, University of Pennsylvania, Philadelphia, PA, United States; ^3^Department of Psychology and Neuroscience, Duke University, Durham, NC, United States

**Keywords:** stress, cortisol reactivity, expressive writing, Trier Social Stress Test (TSST), sustained attention, psychosocial stress, cortisol

## Abstract

Acute stress can harm performance. Paradoxically, writing about stressful events—such as past failures—has been shown to improve cognitive functioning and performance, especially in tasks that require sustained attention. Yet, there is little physiological evidence for whether writing about past failures or other negative events improves performance by reducing stress. In this experiment, we studied the effects of an acute psychosocial stressor, the Trier Social Stress Test, on attentional performance and salivary cortisol release in humans. Additionally, we investigated whether an expressive writing task could reduce the detrimental effects of stress, both on performance and physiological response. We found that when individuals were asked to write about a past failure before experiencing a stressor, they exhibited attenuated stress responses. Moreover, those who wrote about a past failure before being exposed to stress also exhibited better behavioral performance. Our results suggest that writing about a previous failure may allow an individual to experience a new stressor as less stressful, reducing its physiological and behavioral effects.

## Introduction

Acute stress can be harmful to performance. In a real world setting, high levels of stress have been known to cause individuals to “choke under pressure,” resulting in suboptimal performance (Beilock and Carr, 2005). “Choking under pressure” has been found to occur in both physical settings, such as high-stakes sporting events (Baumeister, 1984), and in classroom settings, such as during important exams (Beilock and Carr, 2005). Acute stress seems particularly detrimental to performance on tasks that require high levels of sustained attention. In the laboratory, acute stress has been shown to lead to higher rates of error on tasks requiring high levels of sustained attention (Qian et al., 2015).

Because acute stress is harmful to performance, there has been a recent interest in developing stress reduction interventions. Expressive writing, particularly about negative events such as current anxieties, has been shown to lead to *improvements* in performance (DiMenichi and Richmond, 2015), even in a high-stress environment (Ramirez and Beilock, 2011). Although this outcome is counterintuitive, it has been proposed that writing about negative life events leads to positive outcomes because it relieves stress that normally occurs as a result of attempting to inhibit thoughts about these negative life events (Pennebaker, 1997). However, the assertion that stress reduction is the mechanism by which expressive writing about negative events leads to positive outcomes has been understudied. Specifically, writing about failures has been shown to lead to performance improvements on tasks requiring sustained attention (DiMenichi and Richmond, 2015). However, it remains unknown whether writing about failures improves sustained attention because writing about failures reduces stress, or because it allows an individual to perform better *despite* experiencing physiological stress, perhaps by boosting psychological resources (Hemenover, 2003). If writing about past failures prior to an acute stressor reduces stress, then we would observe a reduction in endocrine response to that acute stressor, along with less of an impairment on performance in a sustained attention task following stress.

Acute stress has been shown to activate the hypothalamic-pituitary-adrenal (HPA) axis, resulting in the release of the hormone cortisol in both animals and humans (Hanson et al., 1976). Furthermore, cortisol reliably peaks in the saliva in humans about 20 min after an individual experiences a stressor (Kirschbaum et al., 1993; Dickerson and Kemeny, 2004). However, there is evidence that this response can be buffered with proper stress-reduction interventions (Smyth et al., 2008). Thus, measuring cortisol during and after a laboratory stressor may shed light on whether expressive writing about a negative event prior to stress can act as a stress-reduction intervention.

In the current study, we examined whether expressive writing about a past failure reduces one’s cortisol response to a new psychosocial stressor. We hypothesized that experiencing a psychosocial stressor would result in an increase in cortisol, but writing about a failure before experiencing the stressor would attenuate this cortisol response. We also examined whether expressive writing about past failures improves performance on a task requiring persistent, sustained attention directly after experiencing psychosocial stress. We predicted that stress would harm performance, and that writing about a past failure would attenuate this effect.

## Materials and Methods

### Participants

One hundred and two participants were recruited from the surrounding area of Rutgers University, Newark. Our sample size was based on the performance effect of DiMenichi and Richmond (2015) and the cortisol effect of Kirschbaum et al. (1993). We also ran an additional power analysis based on the averaged effect size from two previous studies that utilized stress interventions (*f*^2^ = 0.28) on cortisol after the Trier Social Stress Test (TSST) (Gaab et al., 2003; Hammerfald et al., 2006). With stress group, writing group, and gender as factors, as well as an error probability of 0.05, this analysis suggests a total sample size of 86 participants. We are therefore confident that our sample size meets adequate power requirements.

The study was approved by the Institutional Review Board at Rutgers University. Participants (mean age = 24.09, *SD* = 7.36; 54% female; 21% white/Caucasian, 25% black/African American, 34% Asian, 1% Native American, 12% “other”) were paid $15 for 1.5 h of participation. All participants completed the study between 1 and 5 p.m., in order to control for circadian fluctuations of cortisol (Dickerson and Kemeny, 2004). Subjects were naive to the purpose of why the saliva samples were being collected. Our saliva testing lab alerted us that two participants produced saliva samples that were contaminated (presumably from food content in the saliva); therefore, their data were not analyzed. Two additional participants’ data were removed from analyses after participants failed to follow instructions (i.e., did not write about their assigned writing prompt).

### Task

#### Procedure Timeline

**FIGURE 1:**
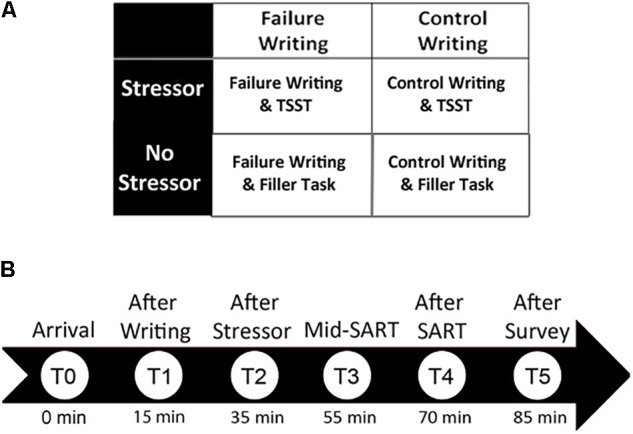
Experimental method. **(A)** Participants were assigned to one of four conditions in which they wrote about a failure or a control topic, and then experienced a stressor or control activity. **(B)** Six salivary cortisol samples were obtained throughout the experiment. Because previous research has found that cortisol peaks about 20 min after a stressor is experienced, all samples represent peak cortisol as a result of the previous event.

Six cortisol samples were obtained throughout the experiment using salivary cheek swabs. After arriving at the laboratory and following giving written consent, participants provided the first salivary cortisol sample (T0), which served as a baseline measurement. Participants were then pseudo-randomly assigned to complete the “failure” or “control” writing manipulation (see below for a detailed description of the writing manipulation). After completing the 10-min writing manipulation, a second salivary cortisol sample was obtained (T1; 15 min elapsed since T0; 20–25 min since arrival). Participants were then pseudo-randomly assigned to complete the TSST (Kirschbaum et al., 1993) or a control task. After completing the TSST or control task, a third saliva sample was taken (T2; 35 min elapsed since T0). Participants then completed the sustained attention to response task (SART; McVay and Kane, 2009; DiMenichi and Richmond, 2015) immediately after completing the TSST to examine the effect of psychosocial stress on attentional performance. Halfway through the SART, a fourth saliva sample was obtained (T3; 55 min elapsed since T0). Finally, the fifth saliva sample was collected at the conclusion of the SART (T4; 70 min elapsed) and the sixth was collected at the conclusion of the survey battery (T5; 85 min elapsed). See **Figure 1** for experimental groups and cortisol timeline.

##### Cortisol collection and assay procedures

Participants were asked to refrain from eating or drinking anything (besides water) at least 1 h prior to participating in the study. Salivary cortisol samples were collected using Salimetrics Oral Swabs. Participants were asked to hold swabs in their cheek for approximately 2 min and to saturate each swab as much as possible with saliva. After this time elapsed, participants were asked to spit the swab into a Salivette vial. Vials were stored in a freezer at -20° before being shipped on dry ice to Salimetrics LLC (Carlsbad, CA, United States), where each sample was assayed twice. The intra-assay variability was 4.66% and the inter-assay variability was 4.47%.

##### Writing manipulation

Participants were pseudo-randomly assigned to complete a writing manipulation adapted from DiMenichi and Richmond (2015). In the “failure” condition, participants saw a prompt on a computer screen that asked them to spend the next 10 min writing about a difficult time in which they did not succeed. They typed their response on the computer. Participants pseudo-randomly assigned to the “control” condition were prompted to write about the plot of a movie they had recently viewed. In order to control for the effect of mood, a follow-up study verified that asking participants to write about a sad movie did not have an effect on attentional performance, suggesting that mood alone is not likely to be the mechanism by which failure writing improves performance on the SART (see Supplementary Material).

Since previous research has found that individual differences within each writing sample (e.g., emotional intensity) can lead to individual differences in outcomes (Harber et al., 1992), two research assistants blind to cortisol and behavioral results read each participant’s writing sample and coded the writing sample for the following five elements: valence (overall positive and negative tone of writing), emotional arousal (i.e., a rating pertaining to how emotional the sample was), compliance with the prompt, relation to oneself, and relation to persistence). Each category was rated with a single score from 1 to 5.

##### Trier Social Stress Test (TSST)

Immediately after completing the writing manipulation, participants assigned to the stress condition completed the TSST (Kirschbaum et al., 1993). The TSST proceeded as follows: the experimenter asked participants about their current career or major, and probed them about their “dream job.” Then, participants were told they would have 6 min to prepare a 5-min speech about why they possess the qualities for their “dream job.” They were also told that they would have to give their 5-min speech in a job-interview format to a “speech expert” while being videotaped and behaviorally analyzed (the “speech expert” was actually a research assistant from the lab). While the participant gave his or her speech, the confederate responded in a cold and unsympathetic manner. If participants did not take the entire 5 min to complete the speech, the speech expert alerted them of the time remaining, and asked them to continue. After 5 min, the speech expert asked the participant to count backwards from 2063 by 13. If the participant made a mistake, he or she was asked to start over from 2063. After 5 min, the speech expert asked the participant to stop.

Participants pseudo-randomly assigned to the control task were probed about their career goals, and then were asked to complete an innocuous personality survey tapping the five OCEAN personality traits for 16 min while alone in a testing room.

##### Sustained Attention to Response Task (SART)

Immediately following the conclusion of the psychosocial stress manipulation or control task, all participants completed a SART (McVay and Kane, 2009; DiMenichi and Richmond, 2015). In this simple “go/no-go” task, participants were told to press the space bar as soon as a letter appeared on the screen, unless that letter was a vowel. Participants were given 2 s to respond to each trial, and the entire SART lasted about 30 min in order to require persistent attention to complete. There were 600 trials, and 20% of trials were vowels (all letters were included except Y).

##### Survey battery

After completing the SART, participants provided information about demographics and daily habits, including smoking habits, contraceptive use, and information about menstrual cycles, since these factors may affect cortisol levels. Furthermore, we distributed a survey battery so that we could explore whether individual differences in cortisol response or SART performance were related to personality traits. The battery included the General Causality Orientations Scale, which assesses intrinsic vs. extrinsic motivations, as well as how much an individual believes circumstances are mostly a matter of luck (Deci and Ryan, 1985); the Connor-Davidson Resilience Scale, which measures individual differences in trait resiliency (Connor and Davidson, 2003); and the Achievement Goal Questionnaire, which examines preference for wanting to achieve goals in order to master a new skill, perform well, or avoid failure (Elliot and Church, 1997). Surveys that examined emotional tendencies included the Beck Depression Inventory-II (Beck et al., 1988), and the Perceived Stress Scale (Cohen et al., 1983), which assesses the extent to which stressors have felt uncontrollable in the last month. The Marlow-Crown Social Desirability Scale was also included to measure any bias in responding on the survey battery (Crowne and Marlowe, 1960). Surveys were completed on a computer via the website Qualtrics (2013) (Provo, UT, United States) and presentation order was randomized by the computer to prevent order effects.

### Analyses

#### Cortisol

##### Preprocessing

Before conducting cortisol analyses, in order to fulfill the requirement for homoscedasticity required for most statistical tests, we examined the skewness of the cortisol measure at each of our 6 timepoints. Across all subjects, every timepoint had a positive skew, averaging 2.01 across all 6 timepoints. Therefore, we performed a power transformation to normalize our cortisol data. Based on a review by Miller and Plessow (2013) that examined the most effective transformations for cortisol time course data, we selected the power transformation x’ = (x^0.26^ - 1)/0.26. After transformation, the skew of all timepoints averaged 0.04. Since there are individual differences in baseline cortisol values, we subtracted each transformed T0 value from the remaining five transformed cortisol timepoints (Mehta and Josephs, 2006). All following analyses use these transformed and baseline-adjusted values.

##### Preliminary manipulation checks

We conducted several preliminary analyses to ensure that our findings were not a result of extraneous variables. First, we conducted a two-way ANOVA examining main effects and an interaction effect of stress group and writing group on the T0 cortisol measurements to ensure that there were no significant differences in baseline cortisol between groups.

We conducted a one-way ANOVA that examined the effect of writing group on cortisol levels at time point T2 (i.e., peak cortisol response since the writing exercise) to examine whether individuals who wrote about a failure showed an increase in cortisol in comparison to individuals who wrote about a control topic. In other words, we sought to ensure that the failure writing exercise did not itself act as an acute stressor.

Since previous studies have shown that gender can influence cortisol levels (Kirschbaum et al., 1995), we also conducted a three-way ANOVA that examined the effect of gender, stress, and writing group on cortisol levels using an area under the curve with respect to increase (AUCi) analysis (Pruessner et al., 2003). We utilized the trapezoidal method, with T0 as our baseline value and points T1–T5 as points in the analysis. Furthermore, since oral contraceptive use has been shown to affect cortisol responsivity (Kirschbaum et al., 1995), among our female participants, we conducted a one-way ANOVA that examined the effect of oral contraceptives on AUCi for female participants. We also tested if smoking habits affected peak cortisol levels by examining whether the number of cigarettes smoked per week significantly correlated with AUCi levels.

##### Main analysis

We conducted a two-way ANOVA examining the effect of writing group and stress group on the AUCi of participants’ cortisol responses. Since we hypothesized that writing about a past failure would attenuate the release of cortisol, we expected to find a significant interaction of stress group and writing group on AUCi levels.

#### Behavior

To examine if reflecting on failures improved performance on the SART after a stressor, we conducted a two-way MANOVA examining the effects of stress group and writing group on errors of commission on the SART (i.e., pressing when the correct answer should be to omit a response), errors of omission on the SART (i.e., failing to press when the correct answer should be to respond), and reaction time on the SART. To examine whether individual differences in cortisol response predicted performance effects, we added AUCi cortisol values as a continuous predictor.

#### Writing Sample Content and Survey Battery

To explore how individual differences related to cortisol response or SART performance, we conducted correlations examining the relationships between (1) baseline cortisol, (2) AUCi of participants’ cortisol levels, (3) SART errors, (4) SART reaction time, (5) scores from all questionnaires in the survey battery, and (6) writing sample ratings.

## Results

### Cortisol Results

#### Results of Manipulation Check Analyses

We conducted several analyses to ensure that our main results were not caused by extraneous variables. To ensure that groups did not differ with respect to cortisol at baseline, we conducted a two-way ANOVA examining the effects of stress group and writing group on baseline cortisol. This analysis did not yield significant main effects [stress: *F*(1,95) < 0.01, *p* = 0.962, $\eta_{p}^{2}$ < 0.01; writing: *F*(1,95) = 0.38, *p* = 0.540, $\eta_{p}^{2}$ < 0.01)] or an interaction effect, *F*(1,95) = 1.95, *p* = 0.166, $\eta_{p}^{2}$ = 0.02. Moreover, the ANOVA that tested whether the two writing groups differed in cortisol level after the writing manipulation did not yield significance, *F*(1,95) = 0.16, *p* = 0.686, $\eta_{p}^{2}$ < 0.01, suggesting that writing about past failures itself did not cause a differential increase in cortisol.

Groups also did not differ significantly from each other in gender, χ^2^ = 0.83, *p* = 0.843, *W* = 0.18, or age, *F*(1,95) = 1.68, *p* = 0.199, $\eta_{p}^{2}$ = 0.02. See **Table 1** illustrating the number of female and male participants in each group. Although we found a significant effect of gender on AUCi values of cortisol, *F*(1,95) = 6.32, *p* = 0.014, $\eta_{p}^{2}$= 0.07, we did not find a significant interaction of stress group and gender on AUCi values, *F*(1,95) = 0.04, *p* = 0.843, $\eta_{p}^{2}$< 0.01, nor did we find a significant interaction of writing × gender, *F*(1,95) = 0.20, *p* = 0.656, $\eta_{p}^{2}$ < 0.01. Our results suggest that although males in our sample tended to have higher cortisol than the females in our sample, these results were not a result of our stress and/or writing manipulations.

**Table 1:** Number of male and female participants across conditions.

	Female	Male
Failure Writing × TSST	15	9
Control Writing × TSST	12	12
Failure Writing × Filler Task	13	11
Control Writing × Filler Task	15	11
**Total**	**55**	**43**

When examining changes in cortisol within our female participants, we did not find a significant effect of oral contraceptives on AUCi values of cortisol, *t*(53) = -0.73, *p* = 0.467, *d* = 0.46. However, only 4 of the 55 women in our sample reported taking oral contraceptives, and all 4 of these women were in the non-stress condition (3 failure writing and 1 control writing). Furthermore, removing these women from our data analysis led to qualitatively similar results. Moreover, the number of participants in each week of menstrual cycle did not differ across condition, χ^2^ = 9.54, *p* = 0.656, *W* = 0.02, nor did day in menstrual cycle significantly correlate with AUCi values across all female participants, *R* = 0.05, *p* = 0.745, or within female stress subjects, *R* = 0.15, *p* = 0.462. See Supplementary Material for Table detailing number of participants in each week of menstrual cycle across conditions.

Groups did not differ from each other in terms of proportion of smokers (failure writing and TSST, *n* = 2; control writing and TSST, *n* = 1; failure writing and filler task, *n* = 1; control writing and filler task, *n* = 1) and non-smokers (failure writing and TSST, *n* = 22; control writing and TSST, *n* = 24; failure writing and filler task, *n* = 23; control writing and filler task, *n* = 24), χ^2^ = 0.17, *p* = 0.876, *W* = 0.08. Furthermore, we did not find a significant correlation between number of cigarettes smoked per week and AUCi levels of cortisol, both across all participants, *r* = -0.18, *p* = 0.083, and within just the stress participants, *r* = -0.231, *p =* 0.113.

#### Writing About Failures Buffers Physiological Stress Responses to the TSST

When examining the effect of stress group and writing group on AUCi values of cortisol, we did not find a significant main effect of stress on AUCi, *F*(1,95) = 2.16, *p* = 0.145, $\eta_{p}^{2}$= 0.02. Moreover, we did not find a significant effect of writing on AUCi values, *F*(1,95) = 0.01, *p* = 0.923, $\eta_{p}^{2}$< 0.01. In line with our hypothesis, we found a significant interaction effect of stress group × writing group, *F*(1,95) = 4.61, *p* = 0.034, $\eta_{p}^{2}$= 0.05. These results suggest that those who wrote about a past failure before undergoing the TSST exhibited significantly reduced cortisol levels.

**FIGURE 2:**
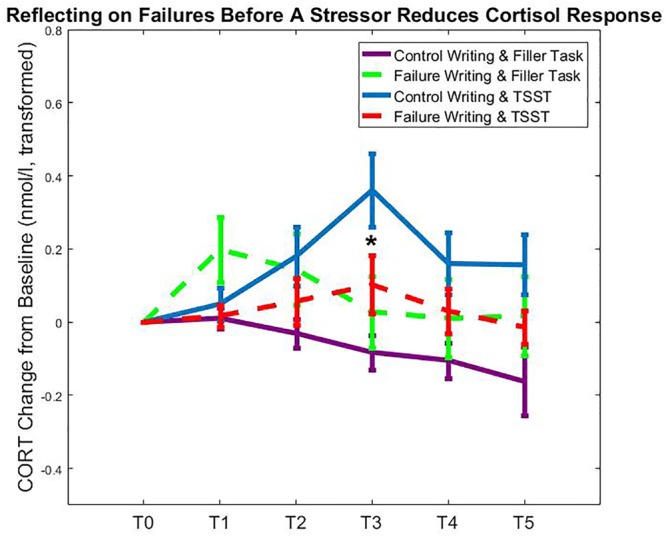
Results. T0 = baseline; T1 = finish writing, 15 min since baseline; T2 = 10 min since stressor onset, 35 min since baseline, expected peak cortisol after writing; T3 = 30 min since stressor onset, 55 min since baseline, expected peak cortisol after stressor; T4 = 45 min since stressor onset, 70 min since baseline; T5 = 60 min since stressor onset, 85 min since baseline. Participants who were subjected to the psychosocial stressor exhibited cortisol increases from baseline (blue line), especially at peak cortisol since stressor conclusion (T3); however, participants who reflected on failures before experiencing the psychosocial stressor exhibited a reduced cortisol response (red line). Participants did not exhibit significant differences at peak cortisol since completing our writing manipulation (T2). Cortisol values represent transformed and baseline-adjusted values (see section “Materials and Methods”).

We also conducted several least-squared differences *post hoc* analyses that examined group differences in AUCi values of cortisol. Specifically, in the control writing groups, the stress manipulation significantly increased cortisol (mean AUCi difference = 19.01, *p* = 0.011). However, this was not the case among participants who wrote about a past failure (mean AUCi difference = 3.56, *p* = 0.636). Thus, our findings suggest that writing about a past failure before undergoing acute stress significantly attenuated the cortisol response to a psychosocial stressor (**Figure 2**). See **Table 2** for AUCi results across groups, and **Table 3** for complete results of the two-way ANOVA.

**Table 2:** Area under the curve from increase (AUCi) values across conditions.

	Mean	*SD*
Failure Writing × TSST	3.57	20.38
Control Writing × TSST	14.35	27.71
Failure Writing × Filler Task	7.13	36.88
Control Writing × Filler Task	–4.66	14.14

**Table 3:** Effects of stress and writing manipulations on AUCi cortisol levels (full two-way ANOVA results).

	AUCi
Corrected model	*F*(1,95) = 2.31
	*p* = 0.081
Stress group	*F*(1,95) = 2.16
	*B* = 0.3.56
	*p* = 0.145
Writing group	*F*(1,95) = 0.01
	*B* = -10.78.
	*p* = 0.923
Stress and writing interaction	***F(1,95) = 4.61^∗^***
	***B = 22.57***
	***p = 0.034***

*B, beta weight; ^∗^*p* < 0.05; ^∗∗^*p* < 0.01.*

### Behavioral Results

#### Writing About a Failure Before Stress Buffers Against Stress’s Effect on Performance

We examined the effect of writing group and stress group on errors of commission, errors of omission, and reaction time in the SART task. We found a significant main effect of writing group on reaction time, *F*(1,96) = 4.89, *p* = 0.029, $\eta_{p}^{2}$= 0.05, whereby writing about failures (regardless of whether the participant experienced a stressor) resulted in significantly slower reaction times on the SART (*M* = 637.83 ms, *SD* = 104.69 ms) compared to those who did not write about a past failure (*M* = 591.03 ms, *SD* = 104.79 ms). We also found a significant interaction of stress group and writing group on errors of commission on the task, *F*(1,96) = 4.55, *p* = 0.036, $\eta_{p}^{2}$= 0.05; participants who wrote about past failures before experiencing a stressor made significantly fewer errors of commission (*M* = 7.75, *SD* = 7.99) than those who did not write about a past failure before experiencing a stressor (*M* = 13.58, *SD* = 7.99). Our results suggest that writing about a past failure resulted in slower reaction times on the SART. Furthermore, writing about a past failure before stress resulted in improved performance. This is consistent with a previously documented speed-accuracy tradeoff in this task (DiMenichi and Richmond, 2015); indeed, here we also found a significant negative correlation between RT and error rates on the SART, *r* = -0.215, *p* = 0.034. See **Table 4** for full MANOVA results and **Table 5** for SART performance across condition.

**Table 4:** Effects of stress and writing manipulations on SART performance (full two-way MANOVA results).

	Commission	Omission	Reaction time
Stress group	*F*(1,95) = 0.40	*F*(1,95) = 3.09	*F*(1,95) = 2.37
	*B* = -2.42	*B* = 7.04	*B* = -39.40
	*p =* 0.527	*p* = 0.082	*p* = 0.127
Writing group	*F*(1,95) = 2.20	*F*(1,95) = 0.54	***F(1,95) = 4.89^∗^***
	*B* = -5.83	*B* = 4.08	***B = 39.95***
	*p* = 0.142	*p* = 0.464	***p = 0.029***
Stress and writing	***F(1,95) = 4.55^∗^***	*F*(1,95) = 0.46	*F*(1,95) = 0.11
interaction	***B = 6.89***	*B* = -3.92	*B* = 13.68
	***p = 0.036***	*p* = 0.500	*p* = 0.747

*^∗^*p* < 0.05.*

**Table 5:** SART performance across stress and writing group.

	Failure Writing × TSST	Control Writing × TSST	Failure Writing × Filler Task	Control Writing × Filler Task
Errors of commission	Mean = 7.75	Mean = 13.58	Mean = 10.17	Mean = 9.12
	*SD* = 6.46	*SD* = 9.12	*SD* = 8.95	*SD* = 7.15
Errors of omission	Mean = 10.21	Mean = 6.13	Mean = 3.17	Mean = 3.00
	*SD* = 22.28	*SD* = 16.19	*SD* = 6.00	*SD* = 6.23
Reaction time	Mean = 618.13	Mean = 578.18	Mean = 657.53	Mean = 603.89
	*SD* = 112.50	*SD* = 107.33	*SD* = 118.93	*SD* = 77.12

We also conducted the same MANOVA described above, and added AUCi as a continuous variable. While previously significant predictors remain unchanged, AUCi did not significantly predict any aspect of SART performance.

#### Individual Differences in Writing Sample Content and Survey Battery

After controlling for multiple comparisons, we did not find any significant relationships across conditions regarding our individual differences measures (both survey and writing content ratings) and our physiological and behavioral results. See Supplementary Material for a correlation table of our survey battery results.

## Discussion

Previous research has suggested that acute stress is harmful to sustained attentional performance (e.g., Qian et al., 2015). However, previous research has also suggested that reflecting about past traumas or current anxieties can improve well-being (Pennebaker, 1997; Niles et al., 2014) and immediate performance (Ramirez and Beilock, 2011). We examined the effect of writing about past failures on cortisol responses to a new psychosocial stressor and sustained attentional performance after stress. We found that when individuals were subjected to the TSST, they exhibited increased cortisol levels, a typical response to a stressful event (Kirschbaum et al., 1995; Dickerson and Kemeny, 2004). However, when individuals wrote about a past failure before experiencing the psychosocial stressor, their cortisol response was attenuated, suggesting that writing about a past failure before experiencing a new stressor may lead to some reduction in one’s physiological experience of stress. Moreover, higher stress responses were associated with poorer performance on a sustained attention task, but writing about failures before a stressor protected against the typical detrimental effect of acute stress on performance. Specifically, while stressed individuals who wrote about a control topic made the most errors of commission, stressed participants who had reflected on failures made the fewest errors of commission.

We did not find evidence that writing about failures alone leads to a significant increase or decrease of cortisol levels, counter to some literature that suggests that writing about past traumas itself affects stress (Pennebaker, 1997). Instead, we propose that writing about failures may make a new stressor seem subjectively less stressful by comparison. Longitudinal data provides support for this claim, as past stressful experiences have been shown to allow an individual to adapt better to a new stressor (Homberg, 2012). Specifically, Stress Inoculation Theory suggests that individuals who have experienced some level of lifetime adversity are more likely to exhibit resilience to a new stressor (Lyons et al., 2009). Furthermore, early life adversity can lead to more adaptive cognition (Frankenhuis and de Weerth, 2013). In the same way that past stressful experiences may allow an individual to adapt to new stressors, writing about a past failure may allow an individual to adapt to a new immediate stressor.

Writing about a failure before experiencing psychosocial stress resulted in reduced cortisol reactivity, as well as better performance on the SART. Although some research has suggested that stress may affect performance in a U-shaped manner (Yerkes and Dodson, 1908; Lupien et al., 2009), others have found that increases in stress result in linear decreases in performance (Domes et al., 2004; Van den Bos et al., 2009). Future work could further examine if there is a linear relationship between stress levels and performance in a sustained attention task.

We also found that writing about a failure resulted in increased reaction time on the SART. All participants in our experiment exhibited a speed-accuracy tradeoff: participants who had the slowest reaction times on the SART also exhibited the best performance. Taken together, these findings support previous claims that writing about a past failure may cause an individual to make slower, more deliberate choices in order to avoid another future failure, resulting in better performance (DiMenichi and Richmond, 2015).

One limitation of the current study is that we did not assess self-reported stress levels throughout the experimental session. A behavioral pilot study we conducted suggested that repeatedly asking individuals about their stress levels after writing about a past failure eliminated the behavioral effects of the writing manipulation. Post-event processing literature suggests that asking participants to repeatedly reflect on stressful feelings about an event can increase negative feelings about that event (Mellings and Alden, 2000), and introspecting on an emotional response may actually change the response (Silvia, 2002; Hutcherson et al., 2005).

Although we found a significant interaction of stress and writing prompt on errors of commission, we did not find a significant main effect of writing group on errors of commission, unlike DiMenichi and Richmond (2015) and our follow-up study described in the Supplementary Material. This could have occurred because the task structure of our task varies from the task structure described in the two other studies: in this study, participants took a 2-min break halfway through the SART to provide a cortisol sample. This break could have improved attention on the SART, resulting in improved performance for all groups, and smaller performance differences between writing groups. Furthermore, in the two other studies, participants wrote about a past failure or control topic and immediately completed a sustained attention task. However, in the non-stress condition in this study, participants completed a filler task before completing the sustained attention task. Perhaps adding this filler task somehow affected performance on the sustained attention task, either because of the task itself or because of experimental timing.

While previous research has suggested that journaling may be beneficial to mental health (Barak and Grohol, 2011), the current study suggests that writing about one’s past failures might not only improve mental health and well-being, but also change the way an individual reacts to future stressors. Perhaps writing about a past failure increases perceived controllability over challenges. It has been shown that increasing perceived control alters the effect of stress on persistence (Bhanji et al., 2016). Future studies might investigate this possibility by assessing or manipulating perceived controllability during stress. Also, although we assessed various traits and tendencies that could contribute to our observed effects, it is unknown if there are other individual differences (e.g., a tendency to disclose, or previous experience with life stressors) that could moderate how strongly writing about a past failure affects stress and performance.

## Conclusion

We found that writing about past failures reduced one’s physiological stress response to a new psychosocial stressor. Most importantly, we found that writing about a past failure before a stressor buffers against decreases in performance that are associated with high levels of stress. In a real-world setting, this information may be valuable to clinicians, as well as educators hoping to improve attentional performance. Since writing about test anxieties has already been shown to protect against the negative effects of stress on performance on a high-stakes exam in a classroom setting (Ramirez and Beilock, 2011), this writing manipulation may be especially valuable to populations who exhibit high levels of performance anxiety.

## Ethics Statement

This study was carried out in accordance with the recommendations of the Rutgers University Institutional Review Board with written informed consent from all subjects. All subjects gave written informed consent in accordance with the Declaration of Helsinki. The protocol was approved by the Rutgers University Institutional Review Board.

## Author Contributions

BD and ET conceived of the experiments and design. BD and CB performed the experiments. BD and KL analyzed the data. All authors contributed to the written manuscript.

## Conflict of Interest Statement

The authors declare that the research was conducted in the absence of any commercial or financial relationships that could be construed as a potential conflict of interest.

## References

[B1] Barak A. Grohol J. M. (2011). Current and future trends in internet-supported mental health interventions. *J. Technol. Hum. Serv.* 29 155–196. 10.1080/15228835.2011.616939

[B2] Baumeister R. F. (1984). Choking under pressure: self-consciousness and paradoxical effects of incentives on skillful performance. *J. Pers. Soc. Psychol.* 46 610–620. 10.1037/0022-3514.46.3.610 6707866

[B3] Beck A. T. Steer R. A. Carbin M. G. (1988). Psychometric properties of the beck depression inventory: twenty-five years of evaluation. *Clin. Psychol. Rev.* 8 77–100. 10.1016/0272-7358(88)90050-5

[B4] Beilock S. L. Carr T. H. (2005). When high-powered people fail working memory and “choking under pressure” in math. *Psychol. Sci.* 16 101–105. 10.1111/j.0956-7976.2005.00789.x 15686575

[B5] Bhanji J. P. Kim E. S. Delgado M. R. (2016). Perceived control alters the effect of acute stress on persistence. *J. Exp. Psychol.* 145 356–365. 10.1037/xge0000137 26726915 PMC4755928

[B6] Cohen S. Kamarck T. Mermelstein R. (1983). A global measure of perceived stress. *J. Health Soc. Behav.* 24 385–396. 10.2307/2136404 6668417

[B7] Connor K. M. Davidson J. R. (2003). Development of a new resilience scale: the Connor-Davidson resilience scale (CD-RISC). *Depress. Anxiety* 18 76–82. 10.1002/da.10113 12964174

[B8] Crowne D. P. Marlowe D. (1960). A new scale of social desirability independent of psychopathology. *J. Consult. Psychol.* 24 349–354. 10.1037/h0047358 13813058

[B9] Deci E. L. Ryan R. M. (1985). The general causality orientations scale: self-determination in personality. *J. Res. Pers.* 19 109–134. 10.1016/0092-6566(85)90023-6

[B10] Dickerson S. S. Kemeny M. E. (2004). Acute stressors and cortisol responses: a theoretical integration and synthesis of laboratory research. *Psychol. Bull.* 130 355–391. 10.1037/0033-2909.130.3.355 15122924

[B11] DiMenichi B. C. Richmond L. L. (2015). Reflecting on past failures leads to increased perseverance and sustained attention. *J. Cogn. Psychol.* 27 180–193. 10.1080/20445911.2014.995104

[B12] Domes G. Heinrichs M. Rimmele U. Reichwald U. Hautzinger M. (2004). Acute stress impairs recognition for positive words—association with stress-induced cortisol secretion. *Stress* 7 173–181. 10.1080/10253890412331273213 15764014

[B13] Elliot A. J. Church M. A. (1997). A hierarchical model of approach and avoidance achievement motivation. *J. Pers. Soc. Psychol.* 72 218–232. 10.1037/0022-3514.72.1.218 10234849

[B14] Frankenhuis W. E. de Weerth C. (2013). Does early-life exposure to stress shape or impair cognition? *Curr. Dir. Psychol. Sci.* 22 407–412. 10.1177/0963721413484324

[B15] Gaab J. Blättler N. Menzi T. Pabst B. Stoyer S. Ehlert U. (2003). Randomized controlled evaluation of the effects of cognitive–behavioral stress management on cortisol responses to acute stress in healthy subjects. *Psychoneuroendocrinology* 28 767–779. 10.1016/S0306-4530(02)00069-0 12812863

[B16] Hammerfald K. Eberle C. Grau M. Kinsperger A. Zimmermann A. Ehlert U. (2006). Persistent effects of cognitive-behavioral stress management on cortisol responses to acute stress in healthy subjects—a randomized controlled trial. *Psychoneuroendocrinology* 31 333–339. 10.1016/j.psyneuen.2005.08.007 16183205

[B17] Hanson J. D. Larson M. E. Snowdon C. T. (1976). The effects of control over high intensity noise on plasma cortisol levels in rhesus monkeys. *Behav. Biol.* 16 333–340. 10.1016/S0091-6773(76)91460-7 818996

[B18] Harber K. D. Pennebaker J. W. Christianson S. (1992). “Overcoming traumatic memories,” in *The Handbook of Emotion and Memory: Research and Theory* ed. Christianson S. A. (Hillsdale, NJ: Lawrence Erlbaum Associates) 359–387.

[B19] Hemenover S. H. (2003). The good, the bad, and the healthy: impacts of emotional disclosure of trauma on resilient self-concept and psychological distress. *Pers. Soc. Psychol. Bull.* 29 1236–1244. 10.1177/0146167203255228 15189585

[B20] Homberg J. R. (2012). The stress-coping (mis) match hypothesis for nature × nurture interactions. *Brain Res.* 1432 114–121. 10.1016/j.brainres.2011.11.037 22169132

[B21] Hutcherson C. Goldin P. Ochsner K. Gabrieli J. Barrett L. F. Gross J. (2005). Attention and emotion: does rating emotion alter neural responses to amusing and sad films? *Neuroimage* 27 656–668. 15946863 10.1016/j.neuroimage.2005.04.028

[B22] Kirschbaum C. Klauer T. Filipp S.-H. Hellhammer D. H. (1995). Sex-specific effects of social support on cortisol and subjective responses to acute psychological stress. *Psychosom. Med.* 57 23–31. 10.1097/00006842-199501000-00004 7732155

[B23] Kirschbaum C. Pirke K.-M. Hellhammer D. H. (1993). The ‘Trier Social Stress Test’–a tool for investigating psychobiological stress responses in a laboratory setting. *Neuropsychobiology* 28 76–81. 10.1159/000119004 8255414

[B24] Lupien S. J. McEwen B. S. Gunnar M. R. Heim C. (2009). Effects of stress throughout the lifespan on the brain, behaviour and cognition. *Nat. Rev. Neurosci.* 10 434–445. 10.1038/nrn2639 19401723

[B25] Lyons D. M. Parker K. J. Katz M. Schatzberg A. F. (2009). Developmental cascades linking stress inoculation, arousal regulation, and resilience. *Front. Behav. Neurosci.* 3:32. 10.3389/neuro.08.032.2009 19826626 PMC2759374

[B26] McVay J. C. Kane M. J. (2009). Conducting the train of thought: working memory capacity, goal neglect, and mind wandering in an executive-control task. *J. Exp. Psychol.* 35 196–204. 10.1037/a0014104 19210090 PMC2750806

[B27] Mehta P. H. Josephs R. A. (2006). Testosterone change after losing predicts the decision to compete again. *Horm. Behav.* 50 684–692. 10.1016/j.yhbeh.2006.07.001 16928375

[B28] Mellings T. M. Alden L. E. (2000). Cognitive processes in social anxiety: the effects of self-focus, rumination and anticipatory processing. *Behav. Res. Ther.* 38 243–257. 10.1016/S0005-7967(99)00040-6 10665158

[B29] Miller R. Plessow F. (2013). Transformation techniques for cross-sectional and longitudinal endocrine data: application to salivary cortisol concentrations. *Psychoneuroendocrinology* 38 941–946. 10.1016/j.psyneuen.2012.09.013 23063878

[B30] Niles A. N. Haltom K. E. B. Mulvenna C. M. Lieberman M. D. Stanton A. L. (2014). Randomized controlled trial of expressive writing for psychological and physical health: the moderating role of emotional expressivity. *Anxiety Stress Coping* 27 1–17. 10.1080/10615806.2013.802308 23742666 PMC3830620

[B31] Pennebaker J. W. (1997). Writing about emotional experiences as a therapeutic process. *Psychol. Sci.* 8 162–166. 10.1111/j.1467-9280.1997.tb00403.x

[B32] Pruessner J. C. Kirschbaum C. Meinlschmid G. Hellhammer D. H. (2003). Two formulas for computation of the area under the curve represent measures of total hormone concentration versus time-dependent change. *Psychoneuroendocrinology* 28 916–931. 10.1016/S0306-4530(02)00108-7 12892658

[B33] Qian S. Li M. Li G. Liu K. Li B. Jiang Q. (2015). Environmental heat stress enhances mental fatigue during sustained attention task performing: evidence from an ASL perfusion study. *Behav. Brain Res.* 280 6–15. 10.1016/j.bbr.2014.11.036 25435315

[B34] Qualtrics (2013). *Qualtrics. com: Qualtric Research Suite*. Provo, UT: Qualtrics.

[B35] Ramirez G. Beilock S. L. (2011). Writing about testing worries boosts exam performance in the classroom. *Science* 331 211–213. 10.1126/science.1199427 21233387

[B36] Silvia P. J. (2002). Self-awareness and emotional intensity. *Cogn. Emot.* 16 195–216. 10.1080/02699930143000310

[B37] Smyth J. M. Hockemeyer J. R. Tulloch H. (2008). Expressive writing and post-traumatic stress disorder: effects on trauma symptoms, mood states, and cortisol reactivity. *Br. J. Health Psychol.* 13 85–93. 10.1348/135910707X250866 18230238

[B38] Van den Bos R. Harteveld M. Stoop H. (2009). Stress and decision-making in humans: performance is related to cortisol reactivity, albeit differently in men and women. *Psychoneuroendocrinology* 34 1449–1458. 10.1016/j.psyneuen.2009.04.016 19497677

[B39] Yerkes R. M. Dodson J. D. (1908). The relation of strength of stimulus to rapidity of habit-formation. *J. Comp. Neurol. Psychol.* 18 459–482. 10.1002/cne.920180503

