# Supplementary material for: Writing About Past Failures Attenuates Cortisol Responses and Sustained Attention Deficits Following Psychosocial Stress
Source: Front Behav Neurosci. 2018 Mar 23;12:45. doi: 10.3389/fnbeh.2018.00045 (PMC5876604; doi:10.3389/fnbeh.2018.00045)
Supplement: Supplementary file 1 [file Data_Sheet_1.docx]

**Supplementary Material**

**Follow-up Study Examining the Effect of Mood**

**Method**

Fifty-one participants were randomly assigned to write about a difficult time in their life in which they did not succeed (failure condition; n=17), the plot of a recently watched movie (control condition; n=17), or the plot of a recently watched sad movie (control-sad condition; n=17). Participants read their randomly assigned prompt on a computer screen, and were asked to write about the topic for 10 minutes. They typed their responses on a computer.

After 10 minutes, participants completed the Sustained Attention to Response Task (SART), a go/no-go task where participants were asked to press the space bar when they saw a letter, unless the letter was a vowel. Participants were given 2 sec to respond to each trial, and the entire SART lasted about 30 minutes, in order to require sustained attention to complete. There were 600 trials, and 20% of trials were vowels (all letters were included except Y). See Figure 2 in the main text for SART illustration.

**Results and Discussion**

A one-way ANOVA examining differences in SART scores between conditions revealed significance (*F*(2,48) = 3.57, *p* = .036). A least-squared differences post-hoc test revealed that participants who reflected on failures exhibited significantly fewer errors of commission on the SART (M = 5.29, SD = 2.89) than participants who wrote about the plot of a recently viewed movie (M = 25.41, SD = 21.08; mean difference = 20.12, *p* = 0.035) and participants who wrote about the plot of a recently viewed sad movie (M = 27.88, SD = 41.70; mean difference = 22.59, *p* = 0.019). There were no significant differences between SART commission error rates for participants who reflected on the plot of a recently viewed movie compared to those who reflected on the plot of a recently viewed sad movie (mean difference = 2.47, *p* =0.791).

Our findings suggest that reflecting on failures does not influence sustained attentional performance through changes in mood alone, since reflecting on a sad movie would also induce sad mood. Instead, our results suggest that something specific about reflecting on failures leads to improvements in sustained attention.

**Menstrual Cycle Across Group for Female Participants**

|  | **Week 1** | **Week 2** | **Week 3** | **Week 4** | **Postmenopausal** |
| --- | --- | --- | --- | --- | --- |
| **Failure Writing x TSST** | 8 | 1 | 3 | 2 | 0 |
| **Control Writing x TSST** | 4 | 3 | 4 | 0 | 1 |
| **Failure Writing x Filler Task** | 4 | 2 | 3 | 3 | 0 |
| **Control Writing x Filler Task** | 5 | 5 | 3 | 2 | 0 |

**Survey Battery Results from Main Experiment**

We examined correlations between our survey measures, and baseline and peak cortisol. Below is a table illustrating these results. SDS = Social Desirability Scale; GCOS = General Causality Orientations Scale; RISC = Connor-Davidson Resilience Scale; AG = Achievement Goal Scale; BDII = Beck Depression Inventor-II; PSS = Perceived Stress Scale. Notably, we found strong relationships between social desirability bias (SDS) and many of our self-report measures.

****significant at p < 0.001*

***significant at p < 0.01*

|  | **Cortisol: baseline** | **Cortisol: AUCi** | **SDS** | **GCOS: Auto** | **GCOS: Control** | **GCOS: Impersonal** | **RISC** | **AG: Performance** | **AG: Mastery** | **AG: Avoidance** | **BDII** | **PSS** |
| --- | --- | --- | --- | --- | --- | --- | --- | --- | --- | --- | --- | --- |
| **Cortisol: baseline** |  | *r = -066.****  *p < 0.001* | *r* = -0.12  *p* = 0.242 | *r* = -0.029  *p* = 0.780 | *r* = 0.13  *p* = 0.209 | *r* = -0.01  *p* = 0.903 | *r* = -0.16  *p* = 0.125 | *r* = 0.18  *p* = 0.077 | *r* = 0.01  *p* = 0.933 | *r* = 0.18  *p* = 0.054 | *r* = 0.22*  *p* =0.031 | *r* = 0.04  *p* = 0.699 |
| **Cortisol: AUCi** |  |  | *r* = 0.10  *p* = 0.314 | *r* = 0.02  *p* = 0.823 | *r* = -0.11  *p* = 0.296 | *r* = 0.02  *p*  = 0.822 | *r* = 0.20  *p* = 0.054 | *r* = -0.16  *p* = 0.108 | *r* = 0.05  *p* = 0.631 | *r* = -0.20*  *p* = 0.047 | *r = -0.24**  *p =0.020* | *r* = -0.10  *p* = 0.379 |
| **SDS** |  |  |  | *r* = 0.03  *p* = 0.810 | *r = -0.28***  *p = 0.005* | *r = -0.39****  *p < 0.001* | *r = 0.43****  *p < 0.001* | *r = -0.12*  *p = 0.248* | *r = 0.27***  *p = 0.007* | *r = -0.27***  *p = 0.006* | *r = -0.25**  *p = 0.012* | *r = -0.26**  *p = 0.019* |
| **GCOS: Auto** |  |  |  |  | *r = 0.28***  *p = 0.006* | *r* = 0.10  *p* = 0.322 | *r = 0.20**  *p = 0.044* | *r* = 0.03  *p* = 0.767 | *r = 0.37****  *p < 0.001* | *r* = -0.11  *p* = 0.268 | *r* = 0.03  *p* = 0.795 | *r* = 0.15  *p* = 0.179 |
| **GCOS: Control** |  |  |  |  |  | *r = 0.48****  *p < 0.001* | *r* = 0.11  *p* = 0.288 | *r = 0.385****  *p < 0.001* | *r* = -0.092  *p* = 0.367 | *r = 0.41****  *r < 0.001* | *r* = 0.05  *p* = 0.619 | *r* = 0.07  *p* = 0.517 |
| **RISC** |  |  |  |  |  |  | *r = -0.23**  *p = 0.025* | *r* = 0.06  *p* = 0.580 | *r = 0.40****  *p < .001* | *r* = -0.12  *p* = 0.250 | *r = -0.37****  *p < 0.001* | *r* = 0.04  *p* = 0.711 |
| **GCOS: Impersonal** |  |  |  |  |  |  |  | *r* = 0.19  *p* = 0.058 | *r* = -0.09  *p* = 0.387 | *r = .52****  *p < 0.001* | *r = 0.29***  *p = 0.004* | *r* = 0.14  *p* = 0.228 |
| **AG: Performance** |  |  |  |  |  |  |  |  | *r* = 0.12  *p* = 0.238 | *r = 0.25**  *p = 0.012* | *r* = -0.14  *p* = 0.157 | *r* = 0.11  *p* = 0.350 |
| **AG: Mastery** |  |  |  |  |  |  |  |  |  | *r* = -0.06  *r* = 0.559 | *r* = -0.03  *p* = 0.799 | *r* = 0.12  *p* = 0.269 |
| **AG: Avoidance** |  |  |  |  |  |  |  |  |  |  | *r = 0.30***  *p = 0.003* | *r* = 0.21  *p* = 0.063 |
| **BDII** |  |  |  |  |  |  |  |  |  |  |  | *r = 0.26**  *p = 0.017* |

**significant at p < 0.05*
